# Supplementary material for: Surface passivation of zero-mode waveguide nanostructures: benchmarking protocols and fluorescent labels
Source: Sci Rep. 2020 Mar 23;10:5235. doi: 10.1038/s41598-020-61856-9 (PMC7089978; doi:10.1038/s41598-020-61856-9)
Supplement: Supplementary file 1 — Supplementary information. [file 41598_2020_61856_MOESM1_ESM.pdf]

Supporting Information for

Surface passivation of zero-mode waveguide nanostructures: benchmarking protocols and fluorescent labels

Satyajit Patra, Mikhail Baibakov, Jean-Benoît Claude, Jérôme Wenger*

Aix Marseille Univ, CNRS, Centrale Marseille, Institut Fresnel, 13013 Marseille, France

** Corresponding author: jerome.wenger@fresnel.fr*

Contents:

1. Fluorescence intensity time traces of green dyes-DNA conjugates inside passivated and unpassivated ZMWs (Figure S1).
2. Apparent fluorescence brightness per molecule enhancement inside ZMWs for different dye-DNA conjugates (Figure S2).
3. Fluorescence lifetime plot of Alexa 546 and Atto 550 labelled DNA in confocal and inside ZMW (Figure S3).
4. FCS correlation functions for different PEG coatings (Figure S4).

S1. Fluorescence intensity time traces of green dyes-DNA conjugates

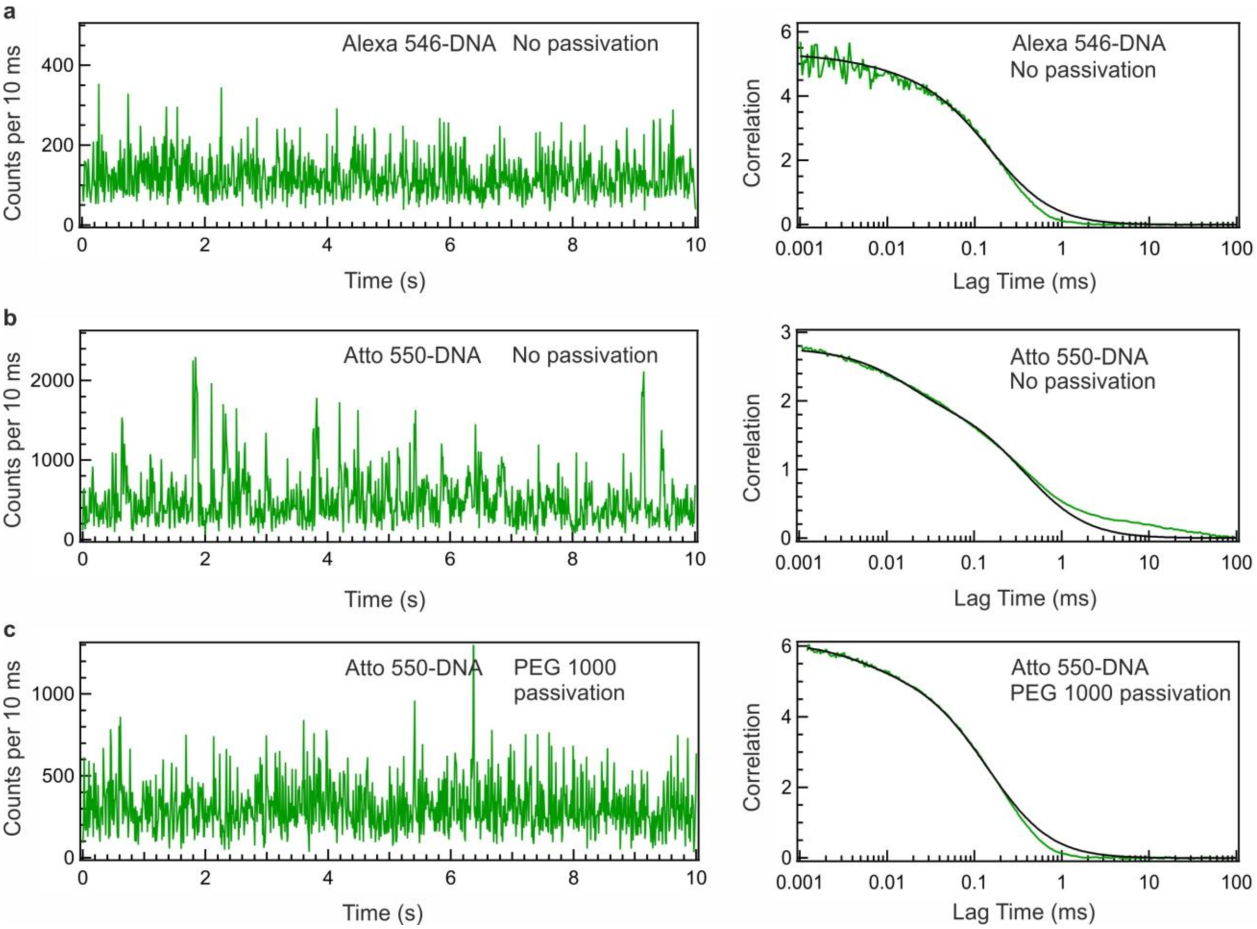

Figure S1: Results obtained from the diffusion of the DNA labelled with green dyes through the attoliter (10^{-18}L) ZMW observation volume. (a) Fluorescence intensity vs time and the corresponding FCS trace obtained for the diffusion of the Alexa 546-DNA through the observation volume of unpassivated ZMW. (b) The fluorescence time trace for Atto 550-DNA inside an unpassivated ZMW shows spikes indicating sticking of the DNA on the ZMW surface, as confirmed by the long diffusing component on the FCS data. (c) Passivation of the ZMW surface with PEG 1000 efficiently eliminates the sticking of Atto 550 labelled DNA sample on the ZMW. The DNA concentration is 100 nM and the ZMW diameter is 110 nm.

S2. Apparent fluorescence brightness per molecule enhancement inside ZMWs for different dye-DNA conjugates

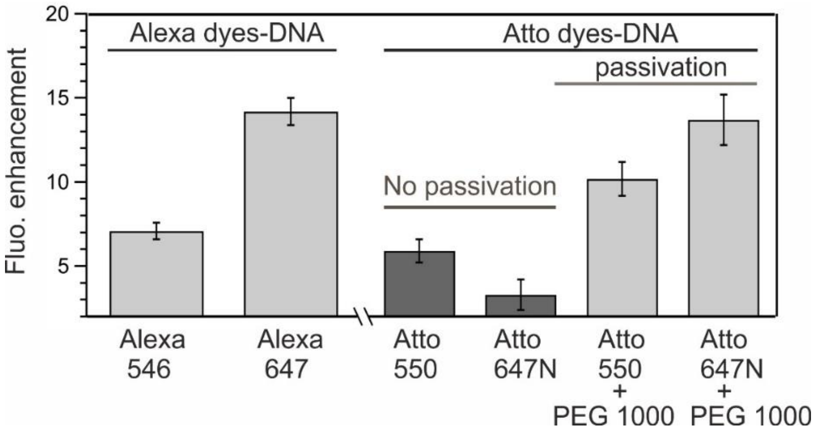

Figure S2: Enhancement factors for the fluorescence brightness per molecule inside 110 nm aluminum ZMWs for different dye-DNA conjugates. The brightness per molecule is computed as the ratio of the average fluorescence intensity divided by the number of molecules estimated by FCS analysis (Fig. 2e), then the enhancement factor corresponds to the ratio of the brightness per molecule in the ZMW to its reference value in the confocal setup. The fluorescence enhancement factors for the Alexa dye labelled DNAs are identical inside passivated and unpassivated ZMWs. The Atto-DNA constructs exhibits higher apparent fluorescence enhancement when the nanoapertures are passivated with PEG 1000 as a result of the misestimate of the number of molecules seen in Fig. 2e when surface sticking occurs.

S3. Fluorescence lifetime plot of Alexa 546 and Atto 550 labelled DNA

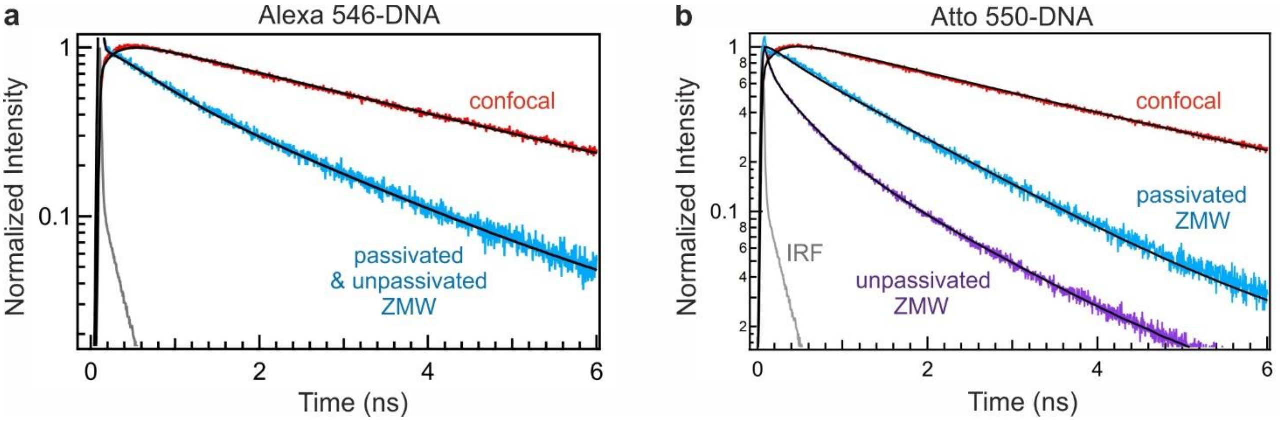

Figure S3: Fluorescence intensity decay plots of Alexa 546 and Atto 550 labelled DNA in the confocal and in the presence of a 110 nm ZMW. For Alexa 546-DNA the results are identical for passivated and unpassivated ZMWs. For Atto 550-DNA, we find a shorter fluorescence lifetime inside unpassivated ZMW than the passivated one, confirming the occurrence of surface sticking inside unpassivated ZMWs.

S4. FCS correlation functions for different PEG coatings

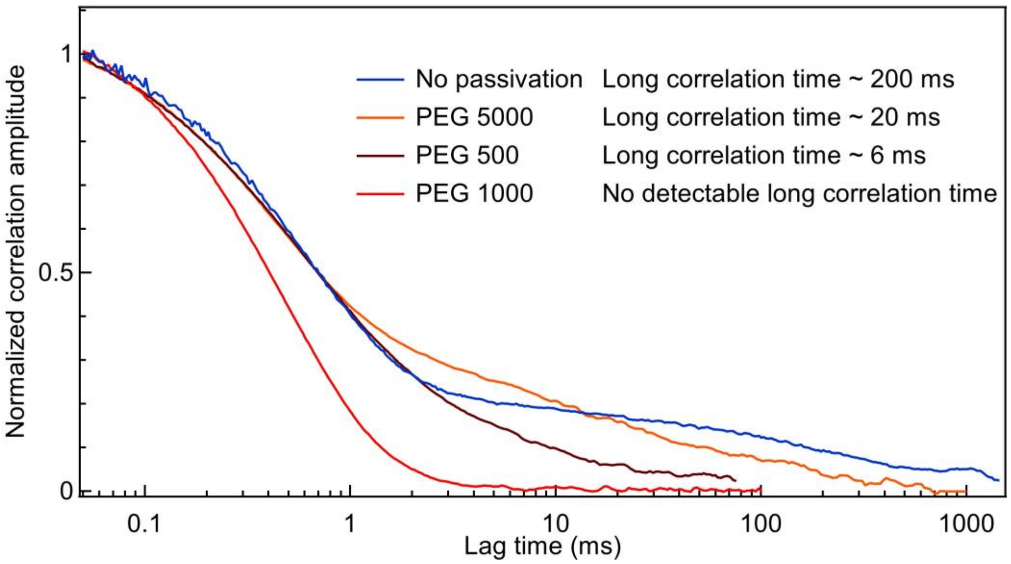

Figure S4: Comparison of normalized FCS correlation functions for different PEG coatings and untreated ZMW surface, in the case of Atto 647N-DNA conjugates in a 110 nm diameter aluminum ZMW (similar to Fig. 4 of the main document). The presence of the long correlation component indicates Atto647N-DNA binding and unbinding (or bleaching) from the ZMW surface. The presence of the PEG coating reduces this long correlation time significantly, which demonstrates that the surface is passivated although sometimes not completely (PEG 500, PEG 5000).
